# Veterinary Medical Society of the University of Pennsylvania

**Published:** 1897-04

**Authors:** John E. Spindler

**Affiliations:** Secretary


### VETERINARY MEDICAL SOCIETY OF THE UNIVERSITY OF PENNSYLVANIA.

THE regular bimonthly meeting was held on Friday evening, March 5, 1897. Meeting called to order at 8 P.M., President F. Klein in the chair.

Minutes of previous meeting read and approved. Mr. J. Hernsheim and Mr. H. Marshall were appointed to look up addresses for sending out letters of inquiry regarding the location of graduates.

Receipts of the evening, $2.40. On motion of Mr. Hernsheim, the sum of $5.00 was appropriated to the Pasteur Monument Fund. The Librarian was authorized to purchase a number of books, among them being Dr. Glass's translation of Müller's *Diseases of the Dog* and Walley's last issue on *Meat inspection.*

Mr. Franz Euge gave a very interesting address on "Horseshoeing," laying special stress on frog-pressure as concerned in diseases of the foot and limb, and strongly recommended the use of the bar-shoe instead of the various pads.

Mr. Zaner reported a case of a five-year-old mare that appeared to be choked. After discussing the case Dr. Bertram gave his opinion that it was meningitis.

President Klein reported a peculiar case of œdema in a cow, extending from both sides of the head over the back and down the posterior limbs, and that the cow was given a simple saline purge and recovered in two days. Mr. Shaw was appointed to look up such cases and enlighten us on the subject at the next meeting.

Critic Shaw reported favorably. Programme for next meeting was read by Mr. Ranck. Meeting adjourned at 10 P.M., to meet March 19, 1897.

The bimonthly meeting was called to order by President Klein, Friday evening, March 19, 1897. Minutes of previous meeting were read and approved.

On motion of Mr. Hernsheim, the chair appointed Messrs. Blount, Gelbert, and Hernsheim as a committee to draw up a set of rules to govern our library and circulation of books therefrom.

Mr. Kirby presented the name of Louis D. Horner, of the class of '98, for membership.

The regular order of business was dispensed with, and Dr. Guy Hinsdale, a member of the Philadelphia Society for the Prevention of Tuberculosis, was then introduced, who addressed the Society on "Climates and their Relation to Disease," and also presented the members each with some valuable pamphlets and tracts on tuberculosis. On motion of Mr. Shaw, the Doctor received the unanimous thanks of the Society for his kindness. On returning to the regular order of business, Mr. Horner was duly elected to membership.

On motion of the Secretary, Dr. Harshberger received the thanks of the Society for reading matter donated by him to our library.

Mr. Blount read a paper on "Oleomargarine." Mr. Hernsheim read a paper on "How Can a Veterinarian Improve His Education?"

Mr. Kirby responded to the referred question, "When, Where, and by Whom was the First Plow Used?" Mr. Shaw responded to the referred question regarding the case reported by the President at the last meeting.

The Secretary read a letter from Dr. Salmon, expressing his personal thanks for the Society's donation to the Pasteur Monument Fund.

The critic of the evening, Mr. Lushington, read a favorable report. Mr. Ranck read the programme for the next meeting. Society adjourned at 10.30 P.M.

JOHN E. SPINDLER,
Secretary.

